# PDBx/mmCIF Ecosystem: Foundation for FAIR Access to Macromolecular Structure Data

**DOI:** 10.1063/4.0000875

**Published:** 2025-10-27

**Authors:** Brinda Vallat

**Affiliations:** RCSB PDB, Rutgers University, Piscataway, NJ, USA

## Abstract

Protein Data Bank (PDB, wwpdb.org, rcsb.org) is the single global repository for three-dimensional structures of biological macromolecules determined experimentally. PDB has been a pioneer in promoting open science and FAIR data practices worldwide. Open access to curated and validated structural biology data provided by the PDB has been crucial for the development of novel protein structure prediction algorithms, elucidated by recent artificial intelligence-based breakthroughs (e.g., AlphaFold2 and RoseTTAFold). Fundamental to these accomplishments are the creation and adoption of data standards. Data standards are technical specifications describing the semantics, logical organization, and physical encoding of data and associated metadata. They are prerequisites for collecting, processing, archiving, and distributing data in a standard format and promote the *FAIR* (*Findable, Accessible, Interoperable and Reusable*) principles emblematic of responsible data management in the modern era. PDB has adopted the PDBx/mmCIF (mmcif.wwpdb.org) data standard that enables FAIR data sharing and facilitates data search, retrieval, and reuse of experimentally-determined macromolecular structures. The PDBx/mmCIF ecosystem also encompasses the IHMCIF data standard that supports archiving of integrative structure models and the ModelCIF data standard that enables archiving of computed structure models. Together, PDBx/mmCIF, IHMCIF, and ModelCIF provide the foundation for comprehensive and FAIR access to structural biology data.

